# Hierarchical clustering of immunohistochemical analysis of the activated ErbB/PI3K/Akt/NF-*κ*B signalling pathway and prognostic significance in prostate cancer

**DOI:** 10.1038/sj.bjc.6605571

**Published:** 2010-03-09

**Authors:** I H Koumakpayi, C Le Page, A-M Mes-Masson, F Saad

**Affiliations:** 1 Centre de recherche du Centre Hospitalier de l’Université de Montréal (CR-CHUM) and Institut du cancer de Montréal, 1560 rue Sherbrooke est, Montréal, Quebec H2L4M1, Canada; 2 Département de médecine, Université de Montréal, Montreal, Quebec H3C3J7, Canada; 3 Département d’urologie, Université de Montréal, Montreal, Quebec H3C3J7, Canada

**Keywords:** prostate cancer, Akt, NF-*κ*B, PTEN, PI3K, outcome

## Abstract

**Background:**

The PI3K/Akt signalling pathway, induced by epidermal growth factor receptor (EGFR) and Her-2, is involved in the constitutive activation of NF-*κ*B in prostate cancer cell lines. In this study, we extended the *in vitro* observation using an *ex vivo* model of prostate cancer tissues and assessed the prognostic significance of the PI3K/Ak/NF-*κ*B signalling determinants.

**Methods:**

We analysed a prostate cancer tissue microarray of 63 patients for the expression of total and activated EGFR, Her-2 receptors and the signalling molecules PTEN, phospho-PTEN, Akt, phospho-Akt and the NF-*κ*B subunit p65. Data were analysed using Spearman's rho test, Kaplan–Meier curves and multivariate Cox regression analysis. In addition, a non-supervised hierarchical clustering analysis was applied to stratify patients according to prognostic groups in terms of risk of recurrence.

**Results:**

The concomitant overexpression of activated EGFR and Her-2 was correlated with the nuclear expression of NF-*κ*B. EGFR, phospho-EGFR, phospho-Her-2, ErbB3 and nuclear NF-*κ*B were associated with the overall biochemical recurrence (BCR) of patients. The non-supervised hierarchical clustering analysis resulted in the separation of patients into five groups according to BCR.

**Conclusions:**

These results validate the previous *in vitro* data on ErbB involvement in NF-*κ*B activation and shows evidence for a significant role of ErbB/PI3K/Akt/NF-*κ*B signalling in the progression of prostate cancer.

Prostate cancer is a leading cause of cancer death in men (Jemal *et al*, 2009). Treatment of prostate cancer depends on its stage at diagnosis. When diagnosed at an early stage, and if the patient is healthy, aggressive therapy is often recommended. However, some early-stage tumours will remain latent and will not require aggressive therapy, whereas others are at risk of progression and need to be treated early. It is unclear what genes or proteins are implicated in the initiation and progression of prostate cancer. Recently, we, along with others, have shown that the p65 subunit of the nuclear factor-*κ*B (NF-*κ*B) transcription factor seems to have a role in the development of prostate cancer and in its progression to an advanced disease (Lessard *et al*, 2003, 2006; Ismail *et al*, 2004; Shukla *et al*, 2004).

Nuclear factor-*κ*B proteins exist as homo- and heterodimers expressed in many cell types but are kept inactive and maintained in the cytoplasm by the I*κ*B family of inhibitors. Classically, the activation of NF-*κ*B requires intracellular signals that combine to activate the trimeric I*κ*B kinase complex (IKK), resulting in the phosphorylation of I*κ*B*α* and its subsequent ubiquitinylation. The net result of this process is translation to the nucleus of NF-*κ*B (Gasparian *et al*, 2002). A constitutive activation of the PI3K/Akt pathway through IKK has been implicated in this activation of NF-*κ*B in the PC-3 hormone-independent prostate cancer cell line (Gustin *et al*, 2001; Gasparian *et al*, 2002; Mayo *et al*, 2002; Le Page *et al*, 2005b). The activation of PI3K results in the translocation of Akt from the cytoplasm to the inner membrane, where Akt is phosphorylated by upstream kinases, PDK and ILK. On the other hand, phosphatase PTEN inhibits the kinase activity of PI3K and thereby suppresses the activation of downstream targets such as Akt. The activation of Akt leads to the phosphorylation of downstream targets such as IKK, GSK3, Bad, mTOR, caspase 9 and the FOXO family of forkhead transcription factors (Vivanco and Sawyers, 2002). More recently, we provided evidence that the epidermal growth factor receptor (EGFR) signalling pathway is implicated as an early event that constitutively activates the PI3K/Akt/NF-*κ*B pathway in PC-3 prostate cancer cells (Le Page *et al*, 2005b).

Epidermal growth factor receptor and Her-2 are members of the ErbB growth factor receptor family composed of four distinct receptors: EGFR/ErbB1, Her-2/ErbB2/c-neu, Her-3/ErbB3 and Her-4/ ErbB4. In PC-3 cells, EGFR and Her-2 overexpression and constitutive activation modulate NF-*κ*B signalling through two distinct mechanisms: one dependent and one independent of the phosphorylation of I*κ*B*α* on ser32/ser36 (Le Page *et al*, 2005b). In PC-3 cells, EGFR, but not Her-2, activates NF-*κ*B through the PI3K/Akt pathway, which induces the phosphorylation of I*κ*B*α* on ser32/ser36 and the subsequent nuclear translocation of NF-*κ*B. Independently, Her-2 also activates NF-*κ*B through the CK2 pathway (Le Page *et al*, 2005b). The two signalling pathways function additively, allowing a stronger activation of NF-*κ*B (Le Page *et al*, 2005a, 2005b). In addition, ErbB3 has been shown to constitutively activate the PI3K pathway in prostate cancer cell lines (Lin *et al*, 1999; Jung *et al*, 2006), which may also lead to the constant NF-*κ*B activation in prostate cancer cells.

In this study, we sought to extend these *in vitro* studies by an *ex vivo* analysis of the determinants of EGFR and Her-2 signalling in NF-*κ*B activation in prostate cancer tissues. For this purpose, we used specific antibodies and immunohistochemistry (IHC) to evaluate the profile of expression of EGFR, Her-2, ErbB3, PI3K, PTEN, Akt, p65 and their phosphorylated forms. The staining with phospho-ErbB3 antibody was too weak to be analysed and was thus not included in this study. Statistical correlations of the protein expression in tissues allowed us to associate signalling events according to the activation of EGFR, Her-2 and PTEN, as well as that of p65. Indeed, this also allowed us to conduct hierarchical analysis to provide a holistic view of pathway interactions and their association with prognosis. This is the first systematic analysis of ErbB receptors and the downstream signalling pathway PI3K/Akt in prostate cancer tissues.

## Materials and methods

### Patient cohort

Paraffin-embedded human primary PCa specimens (collected blocks from 1993 to 2000) from patients who provided informed consent were reviewed. A total of 64 specimens from patients who had a radical prostatectomy were included in our study to create a tissue array. Criteria for the retrospective cohort study to create tissue arrays were the following: (a) no pre- or postoperative treatment was used before biochemical recurrence (BCR) and (b) all cases had a clinical follow-up of at least 3 years or until death. Patients were followed for an average range of 72 months. No age difference was observed between the group of patients who relapsed and the group who did not. Preoperative prostate-specific antigen (PSA) levels were available for 62 patients. Postoperative PSA was available for all patients. Non-failure of PSA was defined as PSA remaining below 0.3 ng/ml after radical prostatectomy. Recurrence-free interval was defined as the time between the date of surgery and the date of first PSA increase above 0.3 ng/ml. The final staging, grading and histological diagnosis were based on the Hôpital Notre-Dame (Montreal, Canada) pathology department report in agreement with the review by an independent pathologist. Ethics approval was obtained from the local IRB committee.

### Tissue arrays, immunohistochemistry, scoring and immunoblotting

Tissue arrays containing a total of 384 cores of prostate tissues (Table 1) were built and used for IHC studies. One normal and two cancerous cores per patient were spotted on one array and a duplicate of this array was made, giving a total of six cores per patient. An expert pathologist reviewed and determined the cancerous, non-neoplastic prostatic epithelium and PIN areas of H&E-stained arrays. However, 11 cores that contained neither cancerous tissues nor non-neoplastic adjacent tissues were not considered for further analysis. The final analysis contained 373 tissue cores representing 63 patients. All patients were represented by at least two cancer cores.

In brief, tissue arrays were deparaffinised in toluene and rehydrated in a gradient of ethanol. Subsequently, endogenous peroxidase activity was quenched by treatment with 0.3% H_2_O_2_/methanol. To unmask antigen, the slides were submerged in 95 °C citrate buffer (10 mM, pH 6.0) or in EDTA buffer (1 mM, pH 8.0) for 15 min. The tissues were blocked for 15 min with a protein-blocking, serum-free reagent (Dako Cytomation Inc., Mississauga, ON, Canada) and incubated with different antibodies for 60 min at room temperature in a humid chamber. The optimal concentration for each primary antibody was determined by serial dilutions. The arrays were then incubated with a secondary biotinylated antibody (Dako Cytomation) for 15 min, followed by incubation with a streptavidin–peroxidase complex (Dako Diagnostics Canada Inc., Mississauga, ON, Canada). Reaction products were developed using diaminobenzidine (brown stain) containing 0.3% H_2_O_2_ as a substrate for peroxidase. Nuclei were counterstained with diluted haematoxylin (blue stain). Tumour sections were inspected at × 20 and × 40 magnification. Epithelial zones were scored according to the staining intensity of the cytoplasm, nucleus or membrane (value of 0 for absence, 1 for weak, 2 for moderate and 3 for high intensity). In cores in which staining was of variable intensity, the average intensity was reported. Each array was independently analysed in a blind study by two independent observers. Interrating correlation was >70%. When strong differences in scoring between the two observers (more than 1 unit per core) occurred, the core was re-evaluated to reach a concordant scoring between the two observers. The average of all cores with cancer from the same patient was used for analysis. No decrease in staining intensity on older paraffin blocks was observed.

The antibodies and conditions used in IHC are summarised in Supplementary Table 1. The specificity of antibodies was previously tested by immunoblotting and showed only one specific band (Supplementary Figure 1). Immunoblotting procedures have been described elsewhere (Le Page *et al*, 2005a, 2005b; Koumakpayi *et al*, 2006). In brief, cells were lysed with cold lysis buffer (10 mM Tris-HCl, pH 7.4, 150 mM NaCl, 1 mM EDTA, 1 mM DTT/1 mM NaF/0.5% NP-40/ 0.5 mM PMSF/0.2 mM sodium orthovanadate/2 *μ*g ml^–1^ of aprotinin, leupeptin and pepstatin), boiled in loading buffer, separated by SDS–PAGE and transferred on a nitrocellulose membrane under refrigerated conditions (60 V for 2 h). The membrane was saturated with 5% milk/PBS/0.1% Tween-20. Immunodetection was carried out as described in the protocol of the ECL kit (Amersham Pharmacia, Little Chalfont, UK). Membranes were incubated for 2 h at room temperature with the specific antibody (0.5–1 *μ*g ml^–1^), washed twice with PBS/0.05% Tween-20 and incubated for another 30 min at room temperature with peroxidase-conjugated antibodies (Santa-Cruz Biotechnology Inc., Santa Cruz, CA, USA).

### Statistical analysis and non-supervised hierarchical clustering analysis

Statistical analysis was carried out with SPSS software 11.0 (SPSS Inc., Chicago, IL, USA). To avoid bias due to a different efficiency of antibody hybridisation on different array slides, we calibrated each slide for interslide comparison. Spearman's rho correlation test (two tailed) was used to estimate the correlation with clinicopathological variables. Survival curves were plotted using the Kaplan–Meier analysis and the log-rank test was used to test for significant differences. Receiver operative characteristic (ROC) curves were used to determine the threshold value for each marker. Cox univariate and multivariate proportional hazard models were used to estimate the hazard ratio for each marker. Multivariate analysis was carried out using a forward stepwise hazard model on univariate analysis required for entry into the model. Only three categorical variables were included in the multivariate Cox regression model to avoid an overfitting situation. These variables were chosen on the basis of their significance in the univariate model and were as follows: surgical margins, Gleason score and pathological stage.

We carried out hierarchical clustering analysis using Genespring software (Silicon Genetics, Santa Clara, CA, USA) on the filtered data set. We used the distance branch of 0.8 with a Pearson correlation as a similarity metric.

## Results

### Expression level and localisation of EGFR, Her-2, ErbB3 and their phosphorylated forms

**Figure 1:**
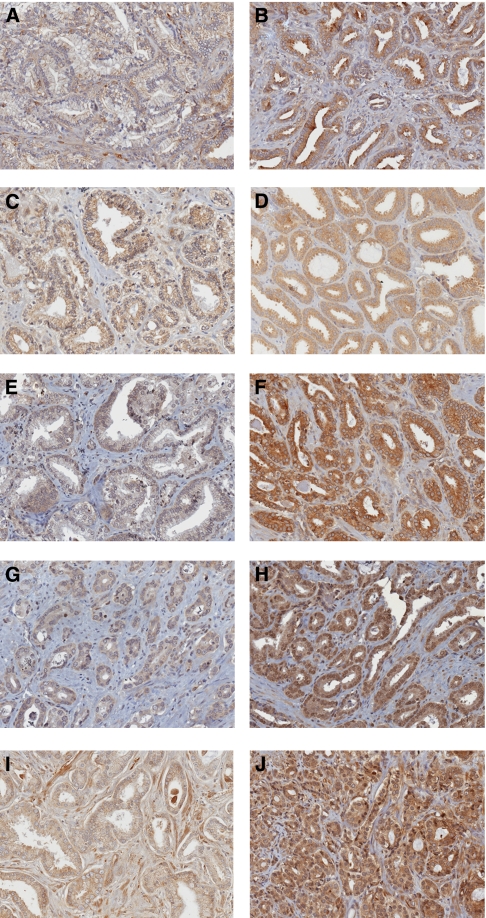
Representative staining for immunohistochemistry of EGFR, Her-2 and ErbB3 in prostate cancer tissues. (**A, C**) Low and moderate EGFR and phospho-EGFR cytoplasmic staining, respectively. (**B, D**) EGFR and phospho-EGFR strong cytoplasmic immunostaining, respectively. (**E, G**) Low and moderate staining for Her-2 and phospho-Her-2, respectively. (**F**) Strong cytoplasmic Her-2 staining. (**H**) Strong cytoplasmic staining with nuclear staining for phospho-Her-2. (**I**) Moderate cytoplasmic ErbB3 staining. (**J**) Strong cytoplasmic and moderate nuclear ErbB3 staining. Each individual marker is indicated. Magnification × 20.

Membrane and cytoplasmic reactivity rates were evaluated in samples of 63 prostate cancer patients (cohort described in Table 1). All cores showed detectable staining for EGFR, phospho-EGFR, Her-2, phospho-Her-2 and ErbB3, mainly localised in the membrane and cytoplasm of cell tissue (Figure 1 and Supplementary Table 2). We did not observe any nuclear staining with EGFR and Her-2, but we observed nuclear localisation of ErbB3 as previously described (Gannon *et al*, 2008) and of phospho-Her-2. Out of 63 patients, 42 showed nuclear ErbB3 presence in cancerous tissues. Only four patients showed weak staining (below 1) for EGFR (Supplementary Table 2). Three of these patients also showed a concomitant weak staining for phospho-EGFR. Similarly, only one patient showed weak staining for Her-2, which was associated with a weak staining of phospho-Her-2. It is noteworthy that only one patient showed a weak phospho-EGFR signal when a strong EGFR signal was observed. The same patient also showed a strong Her-2 expression but a weak phospho-Her-2 staining. In general, a very strong correlation between EGFR and phospho-EGFR staining, and between Her-2 and phospho-Her-2 staining, was found (*r*=0.63 and *r*=0.45, respectively, *P*<0.001, Spearman's rho test; Table 3). Similarly, EGFR staining was also often associated with Her-2 and ErbB3 staining (*r*=0.42 *P*=0.001 and 0.56 *P*<0.001, respectively) and a very strong correlation was obtained between phospho-EGFR and phospho-Her-2 (*r*=0.73, *P*<0.001). A slightly weaker correlation was observed between Her-2 and ErbB3 (*r*= 0.0.38, *P*=0.002).

In 65% of patients (41 of 63), cancer tissues showed an overexpression of EGFR compared with normal tissues, whereas 79 and 81% (50 of 63 and 51 of 63, respectively) of patients showed an overexpression of cytoplasmic Her-2 and ErbB3, respectively (Supplementary Table 2). These results suggest that Her-2 and ErbB3 are overexpressed more often than EGFR in prostate cancer tissues. However, a similar number of patients overexpressed phospho-EGFR and phospho-Her-2 (70 and 73%, respectively).

### Expression and localisation of PI3K/PTEN/AKT pathway members

**Figure 2:**
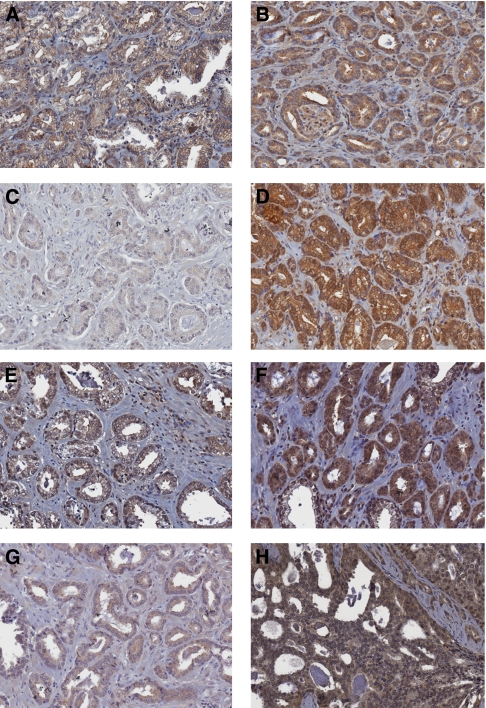
Representative staining for immunohistochemistry of PI3K/Akt/PTEN signalling pathway markers in prostate cancer tissues. (**A, B**) Low and strong cytoplasmic PI3K immunostaining. (**C**) Low cytoplasmic staining for PTEN. (**D**) Strong cytoplasmic staining and nuclear staining of PTEN. (**E**) Phospho-PTEN low cytoplasmic staining. (**F**) Strong cytoplasmic staining with high nuclear staining of phospho-PTEN. (**G**) Moderate cytoplasmic p-Akt staining. (**H**) Strong cytoplasmic staining with nuclear p-Akt (top right corner). Each individual marker is indicated. Magnification × 20.

**Figure 3:**
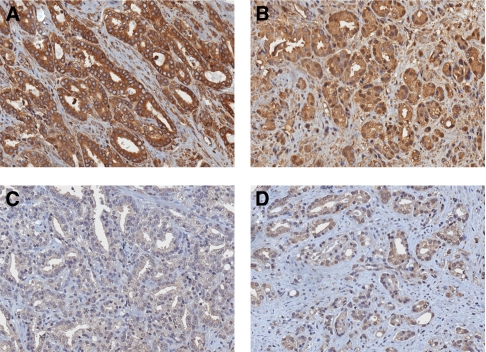
Representative staining for immunohistochemistry of p65/RelA in prostate cancer tissues. (**A**) Strong cytoplasmic staining with no nuclear staining of NF-*κ*B p65 subunit. (**B**) Moderate and strong cytoplasmic staining with nuclear NF-*κ*B p65 subunit. (**C**) Low and moderate phospho-NF-*κ*B p65 subunit. (**D**) Moderate and strong phospho-NF-*κ*B p65 subunit cytoplasmic staining with nuclear staining. Each individual marker is indicated. Magnification × 20.

We also evaluated the activation status of the Akt/PI3K and NF-*κ*B signalling pathway. For this purpose, we immunostained the tissue arrays with antibodies against phospho-Akt, PTEN, phospho-PTEN (Figures 2 and 3). Phospho-Akt and Akt expressions have already been described on this TMA (Le Page *et al*, 2006). In brief, membrane, cytoplasmic and nuclear staining was observed for Akt and for its activated form phospho-Akt. Staining of Akt and phospho-Akt was strongly associated (*r*=0.70, *P*<0.001; Table 3) and both were overexpressed in >60% of patient tumours.

Expression of PTEN was observed in 61 out of 63 prostate cancer patients analysed. A total of 13 patients (20%) had a strong PTEN staining (⩾2.5) and only two patients showed a very weak (<0.5) or an absence of PTEN staining (Supplementary Table 2). Similarly, phospho-PTEN staining was observed in 59 out of 63 patients and was correlated with PTEN staining (*r*=0.40, *P*=0.001, Spearman's test; Table 3). However, 4 out of 10 patients with a weak PTEN staining showed a strong phospho-PTEN staining (Supplementary Table 3). PTEN was mainly expressed in the cytoplasm but a few tissue specimens also showed nuclear staining. Phospho-PTEN was present in both cytoplasmic and nuclear compartments. The expression of phospho-PTEN was decreased in patients with Gleason scores >6 and negatively correlated with Gleason scores (*r*= −0.286, *P*=0.023; Table 2).

In 45 out of 63 cases (71%), PI3K was highly overexpressed in tumour tissues compared with adjacent normal tissues. Only two patients presented a weak PI3K staining (below average intensity 1 on 2 cores). PI3K staining was exclusively cytoplasmic. The intensity of PI3K staining was strongly associated with the expression of PTEN, phospho-PTEN and phospho-Akt (*r*=0.59, *r*=0.70, *r*=0.59, and *P*<0.001, respectively; Table 3).

### Expression and localisation of p65 and correlation with EGFR, Her-2 and ErbB3 pathways

The p65 subunit of NF-*κ*B was expressed in the cytoplasm of all examined prostate cancer tissues (Figure 3 and Supplementary Table 2). Some tissues (*n*=45, 70%) also showed a nuclear staining of p65, suggesting a constitutive activation of NF-*κ*B in these tissues. Phospho-p65 was present in the cytoplasm and a weak staining could be observed in a few cells.

The presence of p65 in the nucleus of prostate cancer tissues was evaluated by the percentage of cells with a nuclear p65 staining. A mean of 2% of cells per tissue showed a p65 nuclear staining. In eight patients, cancer tissues had >10% positive nuclei. Interestingly, the presence of nuclear p65 was increased in patients with Gleason scores of >6, and globally correlated with Gleason scores (*r*=0.28, *P*=0.026 Spearman's test, Table 2). There was no statistical correlation between cytoplasmic and nuclear p65 (*r*=0.11, *P*=0.37), as well as between phosphorylated p65 and nuclear p65 (Table 3, *r*=0.12, *P*=0.343), even when the staining of phospho-p65 and cytoplasmic p65 strongly correlated (Table 3, *r*=0.53, *P*<0.001).

We also evaluated the correlation between nuclear p65 and EGFR, Her-2 and ErbB3 expression. Surprisingly, the presence of nuclear p65 did not correlate with EGFR, Her-2 or ErbB3, either alone or with any other proteins tested (*r*<0.06, *P*>0.05) when continuous values were considered. However dichotomised values of nuclear p65 (high and low) did correlate with combined EGFR and Her-2 (*r*=0.25, *P*=0.047).

### Correlation with clinical parameters

We evaluated the correlation between a strong staining of each antibody and the clinical parameters available for the patient cohort. No correlation was observed between any staining and metastatic spread, perineural infiltration or age (Table 2). In contrast, a strong staining of phospho-EGFR, phospho-Her-2 and nuclear NF-*κ*B correlated with disease stage (*r*=0.32, 0.36, 0.32 and *P*=0.012, 0.004, 0.011, respectively). A trend towards significance was observed for EGFR and ErbB3 (*r*=0.21 0.22, *P*=0.09 and 0.08, respectively). Both phospho-PTEN and nuclear p65 correlated with the Gleason score (*r*=–0.29, *P*=0.023 and *r*=0.28, *P*=0.026). Only phospho-PTEN was significantly and inversely correlated with death due to prostate cancer (*r*=−0.32, *P*=0.011) and a positive trend was observed with nuclear p65 staining (*r*=0.24, *P*=0.06) and phospho-Akt (*r*=0.22, *P*=0.08; Le Page *et al*, 2006). However, the significance of this result should be taken in consideration with the relatively low mortality rate (*n*=9) in this cohort of radical prostatectomy patients.

### Association with biochemical recurrence

**Figure 4:**
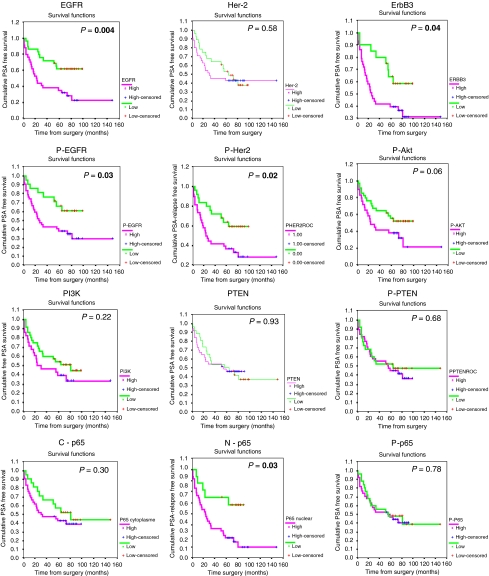
Kaplan–Meier analysis of the ErbB/PI3K/Akt/NF-*κ*B signalling pathway. Kaplan–Meier curves of BCR-free survival in 63 patients with prostate cancer. Each individual marker is indicated in the graph. Significance (*P*) is indicated by log rank.

To evaluate the association between the expression of each protein and the BCR of prostate cancer patients in our cohort, we performed Kaplan–Meier curve analysis coupled with log-rank test and univariate Cox regression analysis. Figure 4 shows the Kaplan–Meier curves obtained. Table 4 shows regression analysis results. Of the candidates listed, EGFR, ErbB3, phospho-EGFR, phospho-Her-2 and nuclear NF-*κ*B were associated with the overall BCR. Phospho-Akt showed a strong trend to associate with BCR (*P*=0.058). In addition, phospho-Akt and PI3K were significantly associated with early BCR at 2 years but not with overall BCR (*P*=0.03 and *P*=0.02, respectively, data not shown). EGFR staining was the most significant event associated with BCR (log rank *P*=0.004 and HR=2.75, *P*=0.05). No combination of proteins, either phosphorylated receptors or signalling proteins, was more significant than EGFR alone (data not shown). In addition, only EGFR was statistically significant in multivariate Cox regression analysis when Gleason score, margin status and pathological stage were considered in the model (*P*=0.03, CI 95% 1.07–4.84).

**Figure 5:**
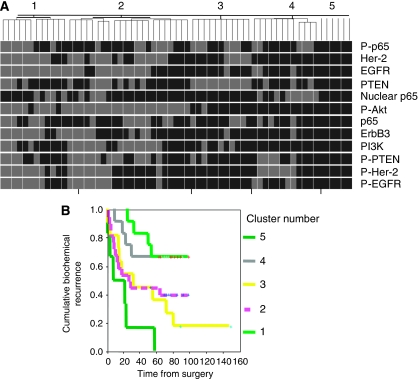
EGFR, Her-2 and PI3K/Akt/NF-*κ*B signalling pathways in an unsupervised hierarchical analysis and association with outcome. (**A**) Unsupervised hierarchical clustering. Each column represents a patient. Each row represents a marker staining as indicated on the left side. Grey colour represents weak marker staining, black represents strong marker staining. (**B**) Kaplan–Meier curve analysis. The five groups of patients identified by unsupervised clustering (clusters 1, 2, 3, 4 and 5 in Figure 4A) were analysed using Kaplan–Meier analysis for biochemical recurrence-free survival. *P*=0.003, significance (*P*) is indicated by log rank.

Using Kaplan–Meier and univariate Cox regression analysis, we also evaluated whether a combination of all markers could be more predictive of BCR than EGFR alone. We used an unsupervised hierarchical clustering analysis to organise patient core staining. This method separated patients into five groups (Figure 5A). A Kaplan–Meier analysis was performed to assess the association of these patient groups and BCR (Figure 5B). Indeed, the unsupervised analysis revealed prognostically relevant patient groups (*P*=0.003, log rank). Interestingly, this combination of multiple markers does not correlate with any clinicopathological parameters and suggests that the combination may provide a new, clinically useful, independent prognostic parameter. Indeed, in Cox multivariate analysis, when clinical variables (Gleason score, margin status and pathological stage) were taken in the model, only EGFR and the multiple marker combination remained an independent predictive variable of BCR (Table 5).

### Association with overall survival

No significant association of the proteins tested was obtained, with the exception of p-PTEN (Table 4, HR=0.02, *P*=0.046). No significant association of p-PTEN and survival was obtained in multivariate analysis. However, the significance of this result should be taken into consideration with the relatively low mortality rate (*n*=9) in this cohort of radical prostatectomy patients.

## Discussion

In prostate cancer cells, the sequential signalling events involved in the constitutive activation of NF-*κ*B have been analysed *in vitro* using cell lines (Sumitomo *et al*, 1999; Suh and Rabson, 2004; Le Page *et al*, 2005a, 2005b). Previously, it has been shown that in the hormone-independent PC-3 cell line, EGFR and Her-2 synergistically induce the activation of NF-*κ*B through two distinct signalling pathways (Le Page *et al*, 2005a). Using selective tyrphostin inhibitors, it has been shown that EGFR activates NF-*κ*B through a mechanism involving the phosphorylation of I*κ*B*α* on serine 32/36, thereby altering the nuclear translocation of p65. On the other hand, Her-2 activates a signalling pathway that does not affect the phosphorylation of I*κ*B*α* on N-terminal serines 32/36. In this study, we attempted to extend these observations to confirm whether EGFR and Her-2 are involved in the constitutive activation of PI3K/Akt and NF-*κ*B signalling pathways in prostate cancer tissues. We found that expressions of EGFR and Her-2 are highly correlated, which makes interpretation difficult for each individual marker on the signalling pathway. Nevertheless, EGFR, Her-2 and ErbB3 alone were correlated with the activation of Akt but not with the nuclear expression of p65. However, combined EGFR and Her-2 significantly correlated with the presence of nuclear p65, supporting our *in vitro* observation in PC-3 cells showing a synergistic effect of EGFR and Her-2 on NF-*κ*B activation (Le Page *et al*, 2005b). This is the first time that a correlation between ErbB signalling and NF-*κ*B activation has been assessed and validated in cancer tissues.

We observed that proteins associated with cell signalling, such as PTEN, p-PTEN, p-Akt and PI3K, correlated with the expression level of EGFR, Her-2 and ErbB3 receptors (Table 3). The expression of PI3K was also strongly associated with PTEN and p-PTEN expression. One could hypothesise that when more PI3K is present in the cell, more PTEN is required to deactivate this kinase. Surprisingly, we also observed a direct correlation between p-Akt and PTEN, although this did not reach significance (*P*=0.07). A similar observation has already been reported in prostate cancer tissues, although the reason for this remains unclear (Bedolla *et al*, 2007; Jendrossek *et al*, 2008) and contrasts with *in vitro* cell line observations in which PTEN expression is inversely correlated with activation of PI3K and Akt. Here, we also observed a positive correlation between p-Akt and p-PTEN. A possible explanation is that, although PTEN would be normally expected to downregulate p-Akt through PI3K, this is counterbalanced by a concomitant inhibition of PTEN by higher levels of pPTEN, which is itself functioning as a negative regulator, thus allowing the observed higher levels of PI3K and pAkt seen in tissues. This hypothesis would require further experimentation for validation.

We also found that EGFR, Her-2 and ErbB3 are strongly correlated and associated with the activation of phospho-Akt, which suggests that these receptors share similar signalling pathways as reported by *in vitro* analysis in cell lines (Yarden, 2001; Jorissen *et al*, 2003). As a consequence, a therapeutic strategy targeting one receptor may not be sufficient, as any receptor may compensate for another's inactivity. Moreover, signalling pathways are complex integrated networks, which render single therapeutic strategies less effective. A supporting argument for this hypothesis is that the activities of EGFR antagonists have not been impressive as a single agent. Numerous clinical trials are currently underway in cohorts of patients with breast and prostate carcinoma to investigate new agents capable of blocking multiple family members such as lapatinib (Bublil and Yarden, 2007). Our study suggests that such inhibitors may be useful in prostate cancer patients.

The present study also analysed and compared the prognostic value of the total expression of EGFR, Her-2 and ErbB3 receptors, as well as their activated forms in a series of primary prostate cancer specimens. The downstream signalling molecules PI3K, PTEN, phospho-PTEN and activated Akt were also analysed. A number of previous studies have already reported the association of EGFR, Her-2 and ErbB3 with PSA biochemical recurrence in prostate cancer patients (Visakorpi *et al*, 1992; Kuhn *et al*, 1993; Fox *et al*, 1994; Di Lorenzo *et al*, 2004; Hernes *et al*, 2004; Zellweger *et al*, 2005; Shah *et al*, 2006; Schlomm *et al*, 2007). In line with these previous studies, we found that EGFR and ErbB3 (Di Lorenzo *et al*, 2002; Hernes *et al*, 2004; Schlomm *et al*, 2007) are also associated with PSA recurrence. Reports on the association of Her-2 and BCR on survival are controversial (Kuhn *et al*, 1993; Signoretti *et al*, 2000; Di Lorenzo *et al*, 2004; Hernes *et al*, 2004; Edwards *et al*, 2006; Isharwal *et al*, 2008) and are likely because of the different types of cohorts studied. In our study, we found that Her-2 was not associated with BCR. However, the phosphorylated tyrosine 1248 of Her-2 was associated with BCR, suggesting that IHC evaluation of the phosphorylated form is better than the standard Her-2 test, which is not clinically used for prostate cancer patients because of a lack of reliability and association with outcome. Interestingly, similar results have been found in breast cancer tissue (Thor *et al*, 2000; Cicenas *et al*, 2006; Frogne *et al*, 2009) when staining of tyrosine 1221/1222 was compared with that of total Her-2. Further studies in larger cohorts would be necessary to validate this initial observation and determine the validity of phospho-Her-2 staining for clinical use in prostate cancer.

When individually compared with each other, the marker that most significantly correlated with PSA recurrence was EGFR. It showed a hazard ratio similar to nuclear p65. Multivariate analysis suggests that EGFR and p65 are two independent prognostic indicators in prostate cancer tissue, which also supports previous reports analysing these two markers separately. Therefore, EGFR and p65 may be interesting markers to help stratify patients for personalised therapy. However, only EGFR was an independent marker in multivariate analysis when Gleason, margin status and pathological stage were included in the model. As EGFR and p65 seem to be strong markers of recurrence in prostate cancer cells, further studies in larger cohorts would be of major interest to evaluate the clinical relevance of these two markers in prostate cancer tissues.

Analysis of the expression level of PTEN and its inactivated phosphorylated form showed that neither correlated with BCR in patients. This result supports other reports showing no significant association between PTEN expression and biochemical recurrence (Bedolla *et al*, 2007; Jendrossek *et al*, 2008; McCall *et al*, 2008), and also conflicts with other studies showing a significant association between low cytoplasmic PTEN and outcome (McCall *et al*, 2008) in a patient cohort with late relapse (>30 months). Altogether, these results suggest that PTEN may only be a prognosis marker for advanced prostate cancer patients. Surprisingly, we did not notice any correlation between a decreased expression of PTEN and an increased activation of Akt, rather we did find a correlation between PI3K and Akt activation. However, this also confirms the previous results obtained in prostate cancer tissues (Bedolla *et al*, 2007; Jendrossek *et al*, 2008). We found that the inactivation of PTEN, as determined by the detection of N-terminal phosphorylation, was more significantly correlated with the increased activation of Akt. This result suggests that the loss of PTEN expression is not the only event regulating the activation of Akt in prostate cancer tissues. Other upstream molecular events, such as activation of kinases responsible for the phosphorylation of PTEN, may also be involved.

Interestingly, in this study, we applied a non-supervised hierarchical analysis of our set of markers, which identified five prognostically significant groups of patients. The same approach has already been successfully used for stratification of breast cancer tissues in TMAs, allowing the classification of breast cancers into clinically relevant prognostic groups. Cells from the patient group with the highest risk of recurrence (group 5, Figure 4) stained positive for all markers tested, which reinforces the importance of the cellular and molecular interaction of each marker in prostate cancer progression even if individually they do not reach prognostic significance.

In summary, our multimarker analysis provides evidence that EGFR, Her-2 and ErbB3 are involved in the constitutive activation of Akt and NF-*κ*B, which validates, for the first time in prostate cancer samples, previous *in vitro* data obtained in cell lines. The importance of PTEN in the constitutive activation of Akt was not validated in our cohort of patients. In addition, we show evidence that the ErbB/PI3K/Akt/ NF-*κ*B signalling pathway is involved in prostate cancer progression and that a multiple marker approach identifying active signalling pathways may be prognostically more relevant than single markers. Further studies including larger patient cohorts should be investigated to confirm these initial results.

## Figures and Tables

**Table 1:** Description of the prostate cancer patient cohort

Age median (min–max)	62 (49–70)
	
*Stage*	
Stage 2	34
Stage 3	29
	
*Invasion*	
Extracapsular invasion	19
Lymph node invasion	9
Peri-neural infiltration	9
Hormone refractory disease	5
Prostatis	1
	
*Gleason score*	
Gleason 4	7
Gleason 5	14
Gleason 6	14
Gleason 7	18
Gleason 8 and 9	10
	
*Pre-operative PSA*	
<10 ng	35
>10 ng	25
Not available	2
	
*PSA relapse*	
Relapse	35
No relapse	28
	
*Surgical margins*	
Negative	31
Positive	32
Survival	54

Abbreviation: PSA=prostate-specific antigen.

**Table 2:** Spearman's correlation between markers and clinicopathological parameters

***P***	**EGFR**	**P-EGFR**	**Her2**	**P-Her2**	**ERBB3**	**PTEN**	**P-PTEN**	**P65**	**P-p65**	**P65 nucl**	**PI3K**	**CLUSTER**
*LN*												
Correlation coefficient	0.013	0	0.091	−0.04	−0.014	−0.118	**−0.411**	−0.096	−0.132	0.238	−0.177	−0.128
***P***	0.919	1	0.477	0.757	0.914	0.355	**0.001**	0.453	0.301	0.06	0.166	0.319
												
*Metastasis*												
Correlation coefficient	0.271	−0.042	0.144	−0.122	0.074	0.127	−0.122	0.208	0.118	0.171	−0.031	0.075
***P***	**0.032**	0.747	0.259	0.341	0.564	0.322	0.341	0.103	0.357	0.179	0.808	0.559
*N*	63	63	63	63	63	63	63	63	63	63	63	63
												
*PSA*												
Correlation coefficient	0.131	0.032	0.059	0.113	−0.041	−0.294	−0.042	−0.137	−0.142	0.179	−0.135	0.198
***P***	0.316	0.805	0.652	0.384	0.756	**0.022**	0.748	0.292	0.275	0.168	0.3	0.126
												
*Age*												
Correlation coefficient	0.008	−0.033	0.055	0.054	0.079	−0.153	−0.13	−0.069	−0.236	−0.154	0.11	0.098
***P***	0.951	0.795	0.671	0.676	0.538	0.231	0.311	0.593	0.062	0.229	0.39	0.445
												
*Gleason*												
Correlation coefficient	−0.008	0.042	−0.17	0.05	−0.076	−0.09	−0.286	−0.166	0.044	0.281	−0.092	−0.116
***P***	0.95	0.746	0.183	0.696	0.555	0.484	**0.023**	0.194	0.733	**0.026**	0.474	0.367
												
*Invasion*												
Correlation coefficient	0.121	0.318	−0.178	0.392	0.225	−0.152	0.038	−0.122	−0.174	0.304	−0.026	0.022
***P***	0.344	**0.011**	0.163	**0.002**	0.076	0.235	0.767	0.34	0.173	**0.015**	0.837	0.867
												
*Margin*												
Correlation coefficient	0.238	0.382	−0.05	0.5	0.215	−0.051	0.045	−0.09	−0.149	0.374	−0.188	0.1
***P***	0.061	**0.002**	0.699	**0**	0.09	0.69	0.724	0.484	0.243	**0.003**	0.14	0.435
												
*Stage*												
Correlation coefficient	0.214	0.315	−0.071	0.359	0.219	−0.002	−0.032	−0.09	−0.097	0.319	−0.125	0.05
***P***	0.092	**0.012**	0.579	**0.004**	0.084	0.987	0.803	0.483	0.449	**0.011**	0.329	0.699
												
*Grade*												
Correlation coefficient	−0.037	0.012	−0.196	0.014	−0.12	−0.11	−0.307	−0.192	0.084	0.311	−0.06	−0.117
***P***	0.771	0.925	0.123	0.915	0.348	0.391	**0.014**	0.132	0.512	**0.013**	0.639	0.361
												
*Infiltra*												
Correlation coefficient	0.195	0.192	0.091	0.238	0.084	−0.026	−0.04	−0.096	0.053	0.134	−0.082	0.079
***P***	0.126	0.131	0.477	0.06	0.515	0.838	0.757	0.453	0.68	0.295	0.525	0.54
												
*BPH*												
Correlation coefficient	−0.162	−0.046	−0.123	0.061	−0.144	−0.177	−0.005	−0.252	−0.072	0.101	−0.018	−0.013
***P***	0.204	0.721	0.336	0.635	0.261	0.166	0.967	**0.046**	0.577	0.433	0.886	0.92
												
*Death*												
Correlation coefficient	0.195	0	0.091	−0.132	0.084	0.066	−0.318	0.096	−0.04	0.238	0.014	−0.012
***P***	0.126	1	0.477	0.301	0.515	0.608	**0.011**	0.453	0.757	0.06	0.916	0.928
												
*Recurrence*												
Correlation coefficient	0.359	0.272	0	0.281	0.236	−0.074	0.084	0.068	0.019	0.305	−0.163	0.198
***P***	**0.004**	**0.031**	1	**0.026**	0.062	0.562	0.511	0.596	0.884	**0.015**	0.201	0.12

Abbreviations: LN=lymph node infiltration; PSA=prostate-specific antigen before surgery; Invasion=extra-capsular invasion; margin=surgical margin; Infiltration=peri-neural infiltration; BPH=benign hyperplasia; BCR=biochemical recurrence; p65 Cyto=cytoplasmic staining of p65; p65 nuc= nuclear p65 staining; Cluster=cluster of non-supervised hierarchical analysis (combination of marker allowing clustering of patients in five groups); Rho=Spearman's coefficient correlation.

Significant correlations (*P*<0.05) are indicated by bold numbers.

**Table 3:** Spearman's correlation between markers

	**EGFR**	**P-EGFR**	**Her2**	**P-Her2**	**ErbB3**	**PTEN**	**P-PTEN**	**PI3K**	**PAKT**	**P65 Cyto**	**P-p65**	**p65 Nuc**
*EGFR*												
Rho	1	**0.625**	**0.424**	**0.624**	**0.565**	0.222	**0.631**	**0.608**	**0.666**	**0.312**	−0.028	−0.047
*P*		**0**	**0**	**0**	**0**	0.08	**0**	**0**	**0**	**0.013**	0.825	0.717
												
*p-EGFR*												
Rho	**0.625**	1	**0.571**	**0.727**	**0.419**	**0.438**	**0.697**	**0.715**	**0.685**	**0.413**	0.098	0.034
*P*	**0**		**0**	**0**	**0.001**	**0**	**0**	**0**	**0**	**0.001**	0.444	0.791
												
*Her2*												
Rho	**0.424**	**0.571**	1	**0.45**	**0.379**	**0.523**	**0.358**	**0.599**	**0.382**	0.228	0.166	0.017
*P*	**0.001**	**0**		**0**	**0.002**	0	**0.004**	**0**	**0.002**	0.073	0.194	0.896
												
*p-Her2*												
Rho	**0.624**	**0.727**	**0.45**	1	**0.416**	0.197	**0.473**	**0.582**	**0.766**	**0.306**	0.036	−0.06
*P*	**0**	**0**	**0**		**0.001**	0.122	**0**	**0**	**0**	**0.015**	0.778	0.639
												
*ErbB3*												
Rho	**0.565**	**0.419**	**0.379**	**0.416**	1	0.189	**0.483**	**0.579**	**0.497**	0.236	−0.125	−0.146
*P*	**0**	**0.001**	**0.002**	**0.001**		0.138	**0**	**0**	**0**	0.062	0.33	0.254
												
*PTEN*												
Rho	0.222	**0.438**	**0.523**	0.197	0.189	1	**0.323**	**0.592**	0.129	**0.419**	0.232	0.075
*P*	0.08	**0**	**0**	0.122	0.138		**0.01**	**0**	0.313	**0.001**	0.067	0.56
												
*P-PTEN*												
Rho	**0.631**	**0.697**	**0.358**	**0.473**	**0.483**	**0.323**	1	**0.704**	**0.592**	**0.369**	0.158	0.022
*P*	**0**	**0**	**0.004**	**0**	**0**	**0.01**		**0**	**0**	**0.003**	0.215	0.863
												
*PI3K*												
Rho	**0.608**	**0.715**	**0.599**	**0.582**	**0.579**	**0.592**	**0.704**	1	**0.586**	**0.432**	0.208	−0.026
*P*	**0**	**0**	**0**	**0**	**0**	**0**	**0**		**0**	**0**	0.102	0.841
												
*P-AKT*												
Rho	**0.666**	**0.685**	**0.382**	**0.766**	**0.497**	0.129	**0.592**	**0.586**	1	**0.301**	0.038	0.019
*P*	**0**	**0**	**0.002**	**0**	**0**	0.313	**0**	**0**		**0.016**	0.768	0.884
												
*p65 cyto*												
Rho	**0.312**	**0.413**	0.228	**0.306**	**0.236**	**0.419**	**0.369**	**0.432**	**0.301**	1	**0.528**	0.115
*P*	**0.013**	**0**	0.073	**0.015**	0.062	**0.001**	**0.003**	**0**	**0.016**		**0**	0.371
												
*P-p65*												
Rho	−0.028	0.098	0.166	0.036	−0.125	0.232	0.158	0.208	0.038	**0.528**	1	0.121
*P*	0.825	0.444	0.194	0.778	0.33	0.067	0.215	0.102	0.768	**0**		0.343
												
*p65 Nuc*												
Rho	−0.047	0.034	0.017	−0.06	−0.146	0.075	0.022	−0.026	0.019	0.115	0.121	1
*P*	0.717	0.791	0.896	0.639	0.254	0.56	0.863	0.841	0.884	0.371	0.343	

Abbreviations: p65 Cyto=cytoplasmic staining of p65; p65 Nuc=nuclear p65 staining; P-=phospho-; Rho=Spearman's coefficient correlation.

Significant correlations (*P*<0.05) are indicated by bold numbers.

**Table 4:** Cox regression univariate for EGFR/Her-2/ErbB3 and signaling molecules

	**BCR**				**OS**			
			**95% CI**				**95% CI**	
	***P***	**HR**	**Lower**	**Upper**	***P***	**HR**	**Lower**	**Upper**
EGFR	**0.005**	2.752	1.35	5.612	0.279	2.447	0.484	12.364
P-EGFR	**0.036**	2.319	1.055	5.1	0.787	0.82	0.196	3.439
Her2	0.587	1.2	0.621	2.318	0.648	1.396	0.333	5.852
P-Her2	**0.028**	2.267	1.091	4.713	0.344	0.527	0.14	1.986
ErbB3	**0.047**	2.221	1.009	4.889	0.576	1.58	0.318	7.845
PTEN	0.933	1.029	0.532	1.99	0.139	3.502	0.664	18.461
P-PTEN	0.683	1.152	0.583	2.276	**0.046**	0.202	0.042	0.976
PI3K	0.222	1.504	0.782	2.893	0.393	1.777	0.476	6.64
P-Akt	0.064	1.867	0.964	3.612	0.641	0.71	0.168	2.997
p65 Cyto	0.304	1.452	0.713	2.954	0.39	1.997	0.412	9.686
P-p65	0.785	1.098	0.561	2.146	0.646	0.734	0.196	2.751
p65 Nuc	**0.034**	2.786	1.081	7.182	0.349	30.772	0.024	NR
P-EGFR/P-Her2	0.122	0.78	0.568	1.069	0.37	0.738	0.38	1.434
CLUSTER	0.103	1.241	0.957	1.609	0.808	0.928	0.509	1.692

Abbreviations: HR=hazard ratio; BCR=biochemical recurrence; OS=overall survival.

Significant correlations (*P*<0.05) are indicated by bold numbers.

**Table 5:** Cox regression multivariate analysis for EGFR and Cluster

	***P***	**HR**	**95% CI**	
EGFR	**0.034**	**2.271**	1.066	4.836
Gleason	**0.007**	**1.489**	1.115	1.989
Stage	0.503	1.516	0.449	5.116
Margins	0.111	2.722	0.795	9.318
CLUSTER	**0.034**	**1.384**	1.025	1.869
Gleason	**0.011**	**1.489**	1.095	2.024
Stage	0.112	2.528	0.804	7.944
Margins	0.219	2.033	0.656	6.305

Abbreviations: CI=confidence interval; HR=hazard ratio; EGFR=epidermal growth factor receptor.

Significant correlations (*P*<0.05) are indicated by bold numbers.
