# Supplementary material for: Hierarchical clustering of immunohistochemical analysis of the activated ErbB/PI3K/Akt/NF-κB signalling pathway and prognostic significance in prostate cancer
Source: Br J Cancer. 2010 Mar 9;102(7):1163–73. doi: 10.1038/sj.bjc.6605571 (PMC2853085; doi:10.1038/sj.bjc.6605571)
Supplement: Supplementary Figure Legend [file 6605571x2.doc]

**FIGURE LEGENDS**

**Supplementary Figure 1. Antibody specificity**

**A.** Whole cell extracts from prostate cancer cell lines, PC-3, DU145 and LNCaP were immunoblotted with antibodies against PI3K-p85 subunit, PTEN, phospho-PTEN (p-PTEN), NF-κB p65 subunit (p65) and phospho- NF-κB p65 (p-p65). β-actin was used as a loading control. **B.** A breast cancer cell line, MCF-7, treated with control BSA or epidermal growth factor receptor (EGF) for 6 hours and neuregulin 1 (NRG1) for 6 hours were used for EGFR, Her-2 and their activated forms.
