# Supplementary material for: Hierarchical clustering of immunohistochemical analysis of the activated ErbB/PI3K/Akt/NF-κB signalling pathway and prognostic significance in prostate cancer
Source: Br J Cancer. 2010 Mar 9;102(7):1163–73. doi: 10.1038/sj.bjc.6605571 (PMC2853085; doi:10.1038/sj.bjc.6605571)
Supplement: Supplementary Table 1 [file 6605571x3.doc]

**Supplementary Table 1: Antibodies and immunohistochemistry conditions**
